# Plexus anesthesia versus general anesthesia for carotid endarterectomy: A systematic review with meta-analyses

**DOI:** 10.1016/j.amsu.2021.102327

**Published:** 2021-04-19

**Authors:** M.S. Marsman, J. Wetterslev, F. Keus, D. van Aalst, F.G. van Rooij, J.M.M. Heyligers, F.L. Moll, A.Kh. Jahrome, P.W.H.E. Vriens, G.G. Koning

**Affiliations:** a Department of Vascular Surgery, Rijnstate Hospital, Arnhem, the Netherlands; b Copenhagen Trial Unit, Center for Clinical Intervention Research, Rigshospitalet Copenhagen University Hospital, Copenhagen, Denmark; c Department of Critical Care, University of Groningen, University Medical Center Groningen, the Netherlands; d Department of Anesthesiology, Radboud University Medical Center Nijmegen, Nijmegen, the Netherlands; e Department of Neurology, Medical Center Leeuwarden, Leeuwarden, the Netherlands; f Department of Vascular Surgery, Elisabeth-TweeSteden Hospital, Tilburg, the Netherlands; g Department of Vascular Surgery, University Medical Center Utrecht, Utrecht, the Netherlands; h Department of Vascular Surgery, Medical Center Leeuwarden, Leeuwarden, the Netherlands; i Department of Vascular Surgery, ZGT, Hospital Group Twente, Almelo/Hengelo, the Netherlands

**Keywords:** Carotid endarterectomy, Systematic review, Plexus, General, Local anesthesia, Stenosis

## Abstract

**Introduction:**

Traditional carotid endarterectomy is considered to be the standard technique for prevention of a new stroke in patients with a symptomatic carotid stenosis. Use of plexus anesthesia or general anesthesia in traditional carotid endarterectomy is, to date, not unequivocally proven to be superior to one other. A systematic review was needed for evaluation of benefits and harms to determine which technique, plexus anesthesia or general anesthesia is more effective for traditional carotid endarterectomy in patients with symptomatic carotid stenosis.

**Methods:**

The review was conducted according to our protocol following the recommendations of Cochrane and reported according to the Preferred Reporting Items for Systematic Reviews and Meta-Analyses. Searches were updated on the October 1, 2020. We did not find any randomized clinical trial comparing plexus anesthesia and general anesthesia in carotid endarterectomy with patch angioplasty matching our protocol criteria in patients with a symptomatic and significant (≥50%) carotid stenosis.

**Conclusions:**

Based on the current, high risk of bias evidence, we concluded there is a need for new randomized clinical trials with overall low risk of bias comparing plexus anesthesia with general anesthesia in carotid endarterectomy with patch closure of the arterial wall in patients with a symptomatic and significant (≥50%) stenosis of the internal carotid artery.

Protocol unique identification number (UIN): CRD42019139913, (https://www.crd.york.ac.uk/prospero/display_record.php?RecordID=139913);

## Highlights

• No conclusive evidence in plexus versus general anesthesia in carotid endarterectomy.
• We recommend one or more randomized clinical trials on this subject forfilling CONSORT statements.
• Individual patient datasharing is important to increase sample sizes for future reviews.

## 1 Introduction

**Fig. 1:**
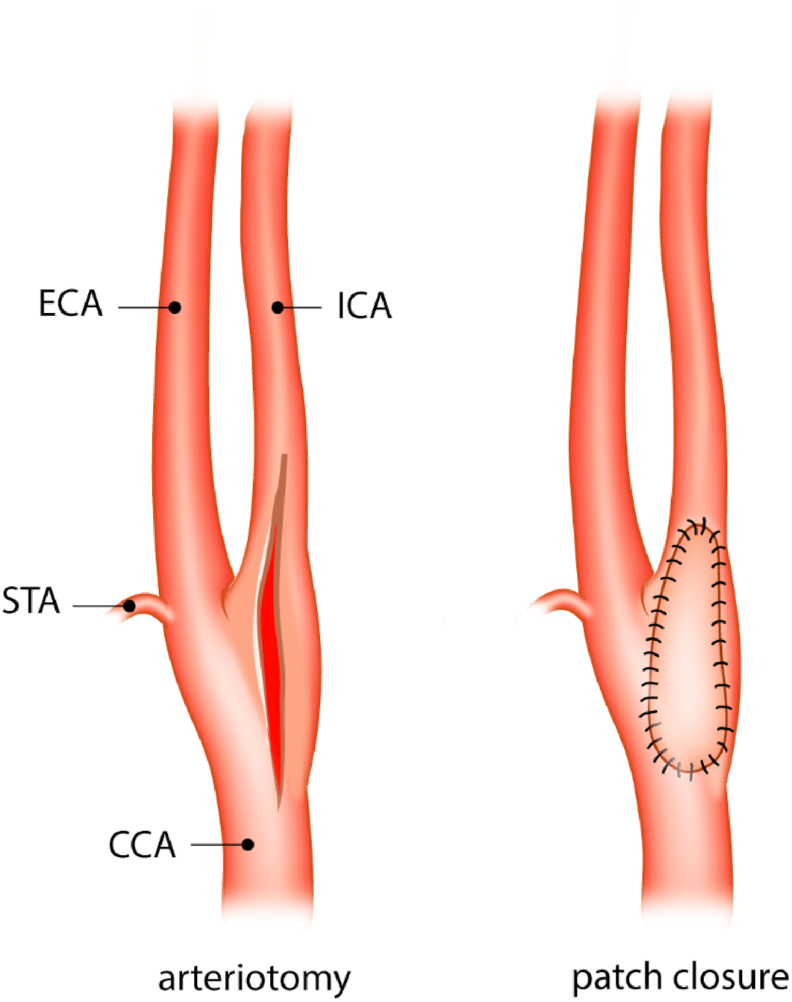
Closure of carotid artery. CCA: common carotid artery, STA: superior thyroid artery, ECA: external carotid artery, ICA: internal carotid artery. A: Longitudinal arteriotomy B: Closure of longitudinal arteriotomy with patch angioplasty.

There is currently no consensus in guidelines which type of anesthesia, plexus anesthesia (PA) or general anesthesia (GA), is best for patients undergoing a carotid endarterectomy (CEA) with patch angioplasty (Fig. 1). Guidelines of both the European Society of Vascular Surgery and the Dutch Society for Vascular Surgery recommend that choice of anesthesia for carotid endarterectomy (PA or GA) be left to the surgical team's preference [[1], [2], [3]]. Patients preferences or instruct ability could also play a role in the choice of anesthetic technique that is used in CEA.

The technique of CEA is previously described by De Bakey [4]. In most Dutch centers GA technique is used for CEA patients. When a patient receives GA for CEA, an opioid, muscle relaxant and an intravenous anesthetic such as propofol is used, followed by intubation and mechanical ventilation [5]. When a patient receives PA for CEA a local anesthetic will be used e.g. ropivacaine [6,7]. PA in short: the patient is in a supine position facing away from the side of the surgery. The anesthetic fluid will be put in place with guidance of anatomical landmarks or ultrasound by an experienced anesthesiologist. Prior to the injection of the anesthetic depot, lidocaine–prilocaine is applied to numb the skin. After skin disinfection the needle will be put in place at the level of the carotid bifurcation and a depot of the anesthetic (e.g. ropivacaine) will be placed underneath the sternocleidomastoid muscle and superiorly and inferiorly along the posterior border of the sternocleidomastoid muscle [[6], [7], [8], [9]]. These landmarks are clearly visible with ultrasound scanning. The carotid sheath can be infiltrated locally using ultrasound by the anesthesiologist or, during surgery, by the vascular surgeon.

Next to the plexus anesthesia, some sedation can be considered to keep the patient comfortable (Richmond Agitation-Sedation Scale 0 to −2) [10] without losing the possibility to test the neurological status (sensory motor skills and verbal testing). This sedation may consist of Dexmedetomidine, the first 10 min at 1 mcg/kg/h, after 10 min around 1/3 of the dosage guided by the heart frequency and or blood pressure. Simultaneously remifentanil is used at 0.1 mcg/kg/min, and after 10 min the dosage is continued at 0.05 mcg/kg/min [6]. Noradrenaline can be used to keep the blood pressure within its desired range to keep the brain adequately perfused [7].

Each type of anesthesia has its (dis)advantages. PA allows real-time direct monitoring (sensory motor skills and verbal testing) compared to indirect monitoring using Transcranial Doppler (TCD) and/or Electroencephalography (EEG) monitoring with GA. TCD and/or EEG monitoring may be normal in 6%–30% of those who develop neurological signs and abnormal in 3%–11% of those who do not develop signs of ischemia [11]. These findings and conclusions of indirect monitoring maybe influenced by the outcome assessors (experience). Propofol can seriously reduce the reliability of perioperative EEG, given the effects it has on brain. Another advantage of PA is that the awake state of the patient does not impair the blood pressure regulation in contrast to GA, which may lower the risk of a peri-procedural stroke [12]. PA is associated with a lower incidence of shunt placement during carotid endarterectomy [13]. The use of a shunt can prevent a perioperative ischemic event. However, it can cause damage to the arterial wall and/or cause an ischemic event [11]. Patients may have less post procedural pain compared with those after GA [14]. The main disadvantage of PA is conversion to GA, which can be necessary when the patient experiences too much pain. PA could also numb the phrenic nerve which can lead to intubation of patients with an already impaired pulmonary function. Other reasons for conversion to general anesthesia can be e.g. claustrophobia, airway obstruction due to cervical hematoma, and shunt-related complications [15].

Incidence of operative complications, such as local hemorrhage, cranial nerve damage, and pulmonary complications has been reported for both PA and GA and showed no differences [16].

Preventive management of (a)symptomatic carotid artery stenosis includes antiplatelet therapy, statins, antihypertensive therapy, diabetic control, as well as lifestyle modifications [[17], [18], [19]]. When a patient shows symptoms, different operation techniques are available and described in literature such as carotid endarterectomy with primary closure, eversion technique and traditional carotid endarterectomy with patch closure. Carotid endarterectomy with patch angioplasty is the preferred guideline treatment for patients with symptomatic stenosis of the carotid artery [20,21], primarily based on the European Carotid Surgery Trial (ECST) and the North American Symptomatic Carotid Endarterectomy Trial (NASCET) [1,4,22,23].

A multicenter, randomized clinical trial (RCT) included 3526 operations in 3526 symptomatic and asymptomatic patients and compared PA with GA and concluded that there was no significant difference between the two techniques for stroke (including retinal infarction), myocardial infarction, and death between randomization and 30 days after surgery [12]. However, the observed difference or lack of difference may or may not be affected by several confounding factors and/or differential use of co-interventions, such as the use of different surgical techniques, selective use of shunting, and variations in materials used for patching [24,25].

Previous conducted systematic reviews with meta-analysis of the randomized trials showed that there was no statistically significant difference between the PA and GA groups in the proportion of patients who had a stroke, died, or had a myocardial infarction within 30 days of carotid endarterectomy [16,26]. These reviews were conducted without Trial Sequential Analysis (TSA). To confirm or reject those meta-analysis results we added TSA and include Grading of Recommendations Assessment, Development and Evaluation (GRADE) assessments of the evidence. We also tried to reduce clinical heterogeneity by comparing only one technique (PA) with one other technique (GA) in patients having carotid endarterectomy with patch angioplasty and also reduce the risk for random error.

## 2 Objective

To determine which technique, PA or GA is more effective for carotid endarterectomy with patch angioplasty of the arterial wall in symptomatic carotid stenosis from the patients’ perspective, it is important that all available evidence is evaluated according to the risks of errors in a systematic review in line with the Cochrane Handbook for Systematic Reviews of Interventions [26,27]. To improve patient centered healthcare, the best possible care should be implemented. Therefore, an updated systematic review with meta-analyses is needed and recommendations can be made for our daily current practice. Updated systematic reviews fuel data for guidelines.

## 3 Material and methods

This review was conducted according to our protocol [28], registered at PROSPERO UIN CRD42019139913, https://www.crd.york.ac.uk/prospero/display_record.php?RecordID=139913 [29] and based on aspects of the recommendations of the ‘Cochrane Handbook for Systematic Reviews of interventions’ [26]. The review is reported according to the Preferred Reporting Items for Systematic Reviews and Meta-Analyses (PRISMA) [30] and Assessing the methodological quality of systematic reviews (AMSTAR) guidelines [31].

### 3.1 Studies

Only trials which evaluate plexus anesthesia versus general anesthesia in carotid endarterectomy with patch angioplasty in adult patients (≥18 years) were included [3]. According to the current guideline [1,22,23], patients with a symptomatic stenosis (≥50 – ≤99%) of the carotid artery were considered. Trials were considered irrespective of language, blinding, outcomes, or publication status.

### 3.2 Experimental intervention

Plexus anesthesia (PA) in carotid endarterectomy with patch angioplasty.

### 3.3 Control intervention

General anesthesia (GA) in carotid endarterectomy with patch angioplasty.

### 3.4 Hypothesis

We wanted to test the null-hypothesis that there is no difference between the two treatments (H0: RRR = 0.00% or RR = 1.00) as well as both the alternative hypotheses (H1a and H1b) that there was a difference (H1a of a 10% RRR or H1b of a 15% RRR) between plexus anesthesia (PA) and general anesthesia (GA) in patients with CEA with patch angioplasty for a symptomatic carotid lesion. For the alternative hypothesis we assumed that patients operated with plexus anesthesia will do better because the neurological status of the patient can be monitored in real time compared with patients operated with general anesthesia in which the surgeon depends on a derived monitoring through TCD and EEG.

### 3.5 Primary outcomes

• Proportion of participants who suffered death (<30 days).
• Proportion of participants with postoperative stroke (<30 days).
• Proportion of participants with one or more serious adverse events; which was defined as: any untoward medical occurrence that results in death, is life threatening, requires hospitalization or prolongation of existing hospitalization, results in persistent or significant disability or incapacity [32].

### 3.6 Secondary outcomes

• Proportion of participants with one or more non-serious adverse events: any untoward medical occurrence in a participant that does not meet the above criteria for a serious adverse event was defined as a non-serious adverse event [32].
• Costs: hospitalization duration, duration of surgical procedure, ICU admission (e.g. blood pressure management).

### 3.7 Exploratory outcomes

• Separately reported serious adverse events.
• Separately reported non-serious adverse events.

The number of patients with one or more complications were evaluated rather than the numbers of events, depending on the availability of data.

### 3.8 Outcome grading

The outcome measures were graded from the patients’ perspective (GRADE working group 2008, Fig. 2) [33]. Examples of serious adverse events: stroke, bleeding, persisting neurological deficits, myocardial infarction, conversion PA to GA due to any cause, patients developing airway obstruction or phrenic nerve palsy and hypertension in need for (intravenous) medication.

**Fig. 2:**
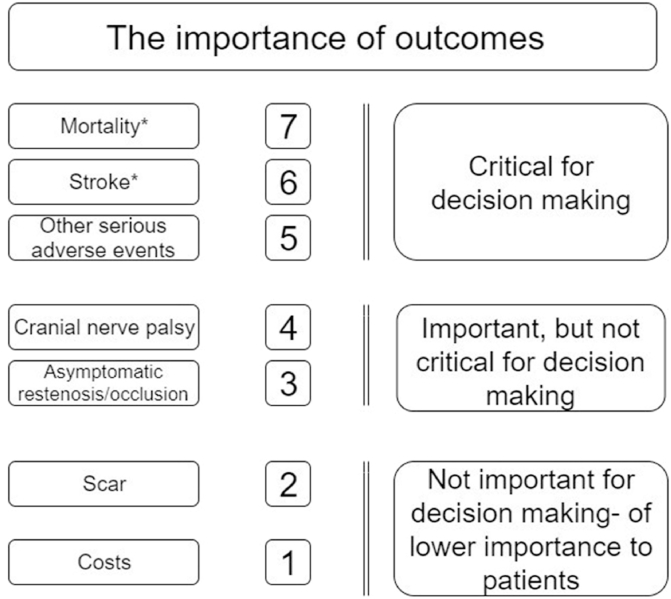
Hierarchy of outcomes from patients' perspective undergoing carotid endarterectomy for symptomatic carotid stenosis (GRADE 2008). * At maximum follow up. Other serious adverse events includes stroke <30 days.

### 3.9 Search strategy

The Cochrane Central Register of Controlled Trials (CENTRAL) in the Cochrane Library, PubMed/MEDLINE and EMBASE databases were searched. References of the identified trials will be searched to identify any further relevant randomized clinical trials. The search strategies are provided in the appendix. Searches will include MeSH descriptors such as “Clinical Trials”, “carotid endarterectomy”, “plexus”, “carotid artery disease”, “anesthesia”, “patch”. We also searched in online trial registries such as ClinicalTrials.gov (https://clinicaltrials.gov/), European Medicines Agency (EMA) (www.ema.europa.eu/ema/), WHO International Clinical Trial Registry Platform (www.who.int/ictrp), and the Food and Drug Administration (FDA) (www.fda.gov) for ongoing or unpublished trials. In addition, we searched Google Scholar (https://scholar.google.nl/) using the terms: anesthesia and/or plexus and/or local anesthesia and/or carotid and/or endarterectomy in the title of the abstract/paper. The overall search was updated on the October 1, 2020.

### 3.10 Data collection

Two authors performed the screening and selected the trials for inclusion, independently. Excluded trials and studies are listed with their reasons for exclusion. When disagreements occurred, a third author was approached to reconcile. The authors extracted the following data when available: type of anesthesia, trial characteristics (year and language of publication, country in which the trial was conducted, year of conduction of the trial, single or multicenter trial, number of patients), patient characteristics (inclusion and exclusion criteria, mean age, mean body mass index and gender, smoking, diabetes mellitus, use of statin and platelet inhibitors), intervention characteristics (general anesthesia, plexus anesthesia, closure by type of patch, use of shunting), co-interventions (conversion to general anesthesia, perioperative transcranial Doppler monitoring, perioperative carotid pressure measurement, electroencephalographic monitoring) and the outcome measures evaluated. If there was any unclear or missing data, the corresponding authors of the individual trials were contacted, at least twice, for clarification.

### 3.11 Risk of bias assessment

Two authors assessed the risks of bias, without masking for trial names, according to the Cochrane Handbook for Systematic Reviews of Interventions [26], including the domains of generation of the allocation sequence, allocation concealment, blinding of participants, personnel, and outcome assessors, incomplete outcome data, selective outcome reporting, and bias risks such as vested interests (financial interest, academic interest or other parties such as the medical industry). Risk of bias components were scored as low, unclear, or high risk of bias.

### 3.12 Statistical methods

Statistical analyses were not performed because no RCT was included. If RCT were found, meta-analyses would be performed according to the Cochrane Handbook for Systematic Reviews of Interventions [26]. The software package Review Manager (RevMan) Version 5.4 was used [34]. Significance levels were adjusted due to multiplicity of several outcomes. The results of each outcome required an adjusted statistical significance level (threshold). An alfa of respectively (0.05/((1 + 3)/2) = ) 0.025 was used for the primary and 0.033 for the secondary outcomes to keep the family wise error rate (FWER) below 0.05 [35,36]. For exploratory outcomes, we considered a *p*-value less than 0.05 as significant, because we view these outcomes as only hypothesis-generating outcomes. For dichotomous variables, the risk ratio (RR) with TSA-adjusted confidence intervals (CI) were calculated. For continuous variables, the mean difference (MD) with TSA-adjusted CI was calculated or the standardized mean difference (SMD).

### 3.13 Trial Sequential analyses (TSA)

Meta-analyses may result in type-I errors and type-II errors due to an increased risk of random error when sparse data are analyzed and due to repeated significance testing when a cumulative meta-analysis is updated with new trials [37,38]. To assess the risk of type-I and type-II errors, TSA could have been used. Detailed TSA description has been published in our protocol.

### 3.14 Grade

We planned to use the summary of findings tables to summarize the results of the trials with overall low risk of bias and for all trials, separately. Reasons for downgrading the quality of the available evidence were: risk of bias evaluation of the included bias domains, publication bias, heterogeneity, imprecision, and indirectness (e.g. length of stay is a surrogate outcome measure) [[39], [40], [41]]. We planned to compare the imprecision assessed according to GRADE with that of TSA [42].

## 4 Results

### 4.1 Study selection

**Fig. 3:**
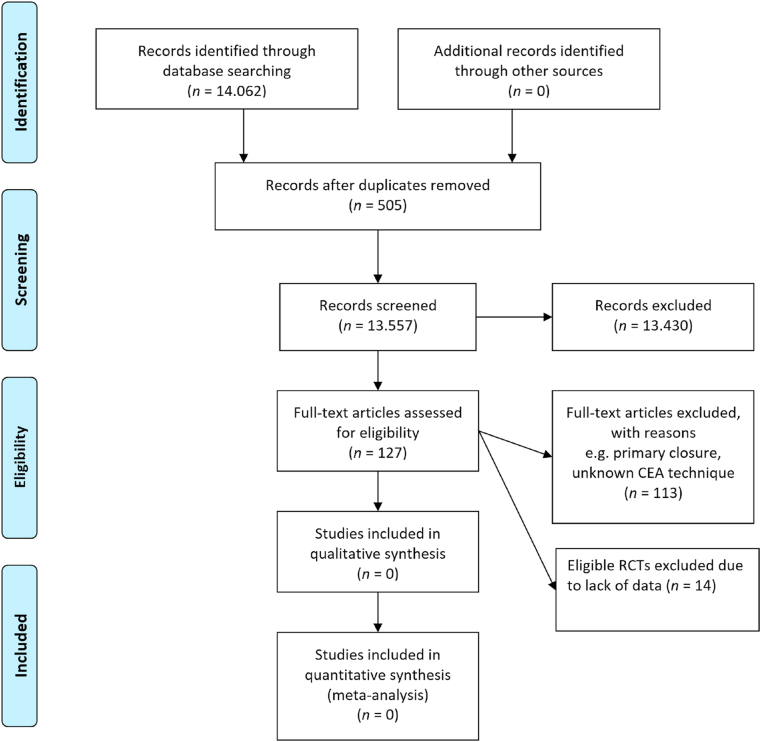
Flow diagram summarizing the search process and results of each phase of the systematic review. https://doi.org/10.1371/journal.pmed1000097.

Altogether the search resulted in 14.062 hits (Fig. 3). In each step of the selection, the publication was included in any case of doubt. Double publications of trial results were considered as one trial. Based on titles and abstracts and removing duplicates 13.935 publications could be excluded. A total of 127 publications remained for full text evaluation from which 113 were excluded based on protocol criteria. Finally, 14 publications were potential trials that could be included. These 14 papers were potentially eligible to include but additional information was lacking. Authors of these studies were contacted at least twice. Despite of our repeated request for data, the response rate was very low. Mazul made clear that their study did not meet our inclusion criteria because of the unknown method of CEA that was used [43]. Luchetti et al. compared asymptomatic patients undergoing plexus anesthesia and plexus anesthesia with general anesthesia in CEA with patch angioplasty [44]. To our best knowledge, no other RCTs conducted met our inclusion criteria.

### 4.2 Patient characteristics and trial designs

The baseline characteristics of the patients were not reported.

### 4.3 Risk of bias

Not performed.

### 4.4 Analysis of outcomes

Not performed.

### 4.5 Trial Sequential Analysis (TSA)

We did not compare the imprecision assessed according to GRADE with that of TSA as planned because we could not perform the primary TSAs as planned in the protocol due to too little statistical information size.

### 4.6 Subgroup analysis

Not performed.

## 5 Discussion

The search in this systematic review lead to 14.062 hits and no randomized clinical trial comparing plexus anesthesia versus general anesthesia in patients with a significant carotid stenosis (≥50%) who underwent carotid surgery (CEA) with patch angioplasty could be included. Authors of other potential eligible trials were contacted at least twice for additional information (such as individual patient data), unfortunately the response rate was very low. This review with extended search showed to date no reliable level of evidence on this subject, fueling the need for a RCT with low risk of bias (level 1b evidence) comparing plexus anesthesia versus general anesthesia in patients with a significant symptomatic carotid lesion (≥50%) who underwent carotid surgery (CEA) with patch angioplasty.

A number of non-randomized clinical trials showed a potential benefit of plexus anesthesia versus general anesthesia in CEA. For example a recent large retrospective study [45] described regional anesthesia compared with general anesthesia in CEA and showed a decreased risk of postoperative pneumonia and a reduced need for perioperative blood transfusions. Difference in 30-day perioperative mortality was not found. Looking at the current evidence of (non) RCTs comparing plexus anesthesia versus general anesthesia in CEA with patch angioplasty it is advised to conduct further investigation with randomization before strong recommendations should be drawn.

The current guideline from ESVS recommend the use of locoregional anesthesia or general anesthesia to the surgical team preference [1]. American Heart Association does not explicitly advise in their guideline which anesthesia should be used [46]. Despite the potential advantages of plexus anesthesia compared with general anesthesia, more than two CEA techniques are being investigated. In most of the current available studies patients were compared whether they had a CEA with patch, or primary closure, symptomatic of asymptomatic stenosis. To our best knowledge the current available level of evidence is 2a at best [27]. Comparing one technique with one other technique lowers the risk of being biased due to the possible influences or several confounding factors and/or different use of co-interventions [26]. Low risk of bias (Level 1a) evidence is presumably scarce, meta analyses are usually seen as trustworthy, but based on Koster et al. only 0.9% of the available meta-analyses of intensive care unit interventions were judged as having low risk of bias (designed and reported according to standards for trustworthy systematic reviews and meta-analyses) [47].

We created a focused review, comparing one technique with one other technique for anesthesia, but also only in symptomatic carotid patients, by excluding asymptomatic patients we may introduce a blind spot to potential information about potential advantages and disadvantages comparing the anesthetic techniques (plexus versus general anesthesia). On the other hand, conducting a broader review may introduce potential confounding by severity of disease. In the ideal world we include all trials comparing plexus versus general in CEA with patch angioplasty in symptomatic patients. Subgroup analysis could have been an option minimizing the risk of confounders.

In this focused review comparing plexus and general anesthesia in symptomatic patients for CEA with patch angioplasty, looking at the current available evidence, we can conclude that more randomized clinical trials with low risk of bias are needed before firm conclusions can be drawn and more reliable recommendations can be made. We may be halfway comparing two different types of techniques (anesthetic: plexus versus general, and, surgical: primary closure versus patch angioplasty) [48], but these futures studies should be with patients suffering from a symptomatic and significant (≥50%) carotid stenosis of the internal carotid artery. A potential obstacle is the size of such randomized clinical trials, to demonstrate a difference in mortality, stroke, and occlusion. The number of patients needed would presumably be high. That is why it is so important that researchers are willing to share individual patient data (Table 1).

**Table 1:** Checklist of recommendations for future randomized clinical trials comparing plexus anesthesia with general anesthesia in patients with a symptomatic and significant (≥50%) stenosis undergoing carotid endarterectomy with patch angioplasty. In an attempt to bridge the information gap, a new trial should at least comprise as many patients as the hitherto largest and that preferably several new trials will be needed with at least as many patients as it takes to produce a boundary break through (boundary for benefit, harm or futility) in the Trial Sequential Analysis, or in the worst case scenario; to close the gap between the required and the presently accrued information size.

Item	Recommendation
To get the evaluation of serious adverse events (SAE) right	Count the number of patients with one or more SAE, and not just the total number of SAE.
To prevent design error	• Protocol based (Published before starting with the trial(s))
	• Standarized surgical technique (e.g. patch or primary closure, type and amount of sutures)
	• Compare ONE experimental intervention (plexus-) to ONE control intervention (general anesthesia).
To avoid bias	Future trials should be in line with the CONSORT statements [49]
To minimize risk of random error	The sample size should exceed e.g. 2000 participants in one or more future trials.
Data sharing	Data sharing is important to increase sample sizes in future trials.
	Trial coordinators should be encouraged to participate in sharing anonymized data upon request review author.
Comparison	Outcome measures critical for decision making according to the GRADE (39).

## 6 Conclusions

Based on the current, high risk of bias evidence, we concluded there is a need for new randomized clinical trials with overall low risk of bias comparing plexus anesthesia with general anesthesia in carotid endarterectomy with patch closure of the arterial wall in patients with a symptomatic and significant (≥50%) stenosis of the internal carotid artery.

## Ethical approval

None.

## Funding

None.

## Author contribution

MSM is the first author of the protocol. MSM and GGK managed the first draft of this manuscript and coordinated the contributions of coauthors. Contributors JW, PWHEV, DA, FGR, JMMH, FLM, AkhJ, FK, and GGK contributed to the design of the study and revised the paper critically. JW, FK, and GGK provided professional and statistical support. All authors read and approved the final version of the manuscript. GGK was initiator and supervisor.

## Research Registration Unique Identifying Number (UIN)

1. Name of the registry: Prospero

2. Unique Identifying number or registration ID: CRD42019139913

3. Hyperlink to your specific registration (must be publicly accessible and will

be checked):

https://www.crd.york.ac.uk/prospero/display_record.php?ID=CRD42019139913

## Guarantor

M.S. Marsman and G.G. Koning

## Declaration of competing interest

JW is a member of the taskforce at Copenhagen Trial Unit to develop theory and software doing TSA, presently available as freeware at www.ctu.dk/tsa.

## Appendix A Supplementary data

The search strategy that was followed in the different online libraries, PubMed/Medline, The Cochrane Library, and Embase is added as supplementary file I. The full key terms and MeSH terms are described. The protocol is added as supplementary file II. The AMSTAR 2 checklist is added as supplementary file III and the PRISMA checklist as supplementary file IV.

Supplementary file I Supplementary file I

Supplementary file II Supplementary file II

Supplementary file III Supplementary file III

Supplementary file IV Supplementary file IV
