# Supplementary material for: Plexus anesthesia versus general anesthesia for carotid endarterectomy: A systematic review with meta-analyses
Source: Ann Med Surg (Lond). 2021 Apr 19;65:102327. doi: 10.1016/j.amsu.2021.102327 (PMC8094902; doi:10.1016/j.amsu.2021.102327)
Supplement: Supplementary file I [file mmc1.docx]

**APPENDIX.**

**Search strategy**

**PubMed Search**

(("Anesthesia"[Mesh] or anesthe* [tiab] OR nerve block* [tiab] OR plexus block* [tiab]) AND ("Blood Vessel Prosthesis"[Mesh] OR "Polyethylene Terephthalates"[Mesh] OR "Polytetrafluoroethylene"[Mesh] OR Polytef[tiab] OR PTFE[tiab] OR TFE[tiab] OR FEP[tiab] OR Tarflen[tiab] OR Fluoroplast[tiab] OR GORE-TEX[tiab] OR Goretex[tiab] OR Teflon[tiab] OR Fluon[tiab] OR Polyethylene Terephthalate[tiab] OR Dacron[tiab] OR Polytetrafluoroethylene[tiab] OR Patch*[tiab] OR Blood Vessel Prosthes*[tiab] OR Vascular Prosthes*[tiab] OR Tissue-Engineered Vascular Graft*[tiab] OR "Angioplasty"[Mesh] OR angioplast*[tiab] OR biopatch*[tiab] OR porcine[tiab] OR bovine[tiab] OR "Endarterectomy, Carotid"[Mesh] OR (carotid[tiab] AND endarterectomy[tiab]) OR cCEA[tiab]))

**EMBASE Search**

(('anesthesia'/exp OR anesthe*:ti, ab OR nerve block*:ti, ab OR plexus block*:ti, ab) AND ('blood vessel prosthesis'/exp OR 'polyethylene terephthalate'/exp OR 'polytetrafluoroethylene covered stent'/exp OR Polytef:ti, ab OR PTFE:ti, ab OR TFE:ti, ab OR FEP:ti, ab OR Tarflen:ti, ab OR Fluoroplast:ti, ab OR ‘GORE-TEX’:ti, ab OR Goretex:ti, ab OR Teflon:ti, ab OR Fluon:ti, ab OR ‘Polyethylene Terephthalate’:ti, ab OR Dacron:ti, ab OR Polytetrafluoroethylene:ti, ab OR Patch*:ti, ab OR ‘Blood Vessel Prosthes*’:ti, ab OR ‘Vascular Prosthes*’:ti, ab OR ‘Tissue-Engineered Vascular Graft*’:ti, ab OR 'angioplasty'/exp OR angioplast*:ti, ab OR biopatch*:ti, ab OR porcine:ti, ab OR bovine:ti, ab OR 'carotid endarterectomy'/exp OR (carotid:ti, ab AND endarterectomy:ti, ab) OR cCEA:ti, ab))

**Cochrane Search**

anesthe* OR nerve block* OR plexus block*

AND

Polytef OR PTFE OR TFE OR FEP OR Tarflen OR Fluoroplast OR GORE-TEX OR Goretex OR Teflon OR Fluon OR Polyethylene Terephthalate OR Dacron OR Polytetrafluoroethylene OR Patch* OR Blood Vessel Prosthes* OR Vascular Prosthes* OR Tissue-Engineered Vascular Graft* OR angioplast* OR biopatch* OR porcine OR bovine OR carotid endarterectomy OR cCEA

**Google Scholar Search**

Search in title: anesthesia AND angioplast OR biopatch OR porcine OR bovine OR endarterectomy OR CEA

Search in Trip, EMA, FDA and WHO ICTRP databases with “Carotid Endarterectomy” in title.
