# Supplementary material for: Plexus anesthesia versus general anesthesia for carotid endarterectomy: A systematic review with meta-analyses
Source: Ann Med Surg (Lond). 2021 Apr 19;65:102327. doi: 10.1016/j.amsu.2021.102327 (PMC8094902; doi:10.1016/j.amsu.2021.102327)
Supplement: Supplementary file II [file mmc2.pdf]

International Journal of Surgery Protocols 19 (2020) 1–7

Contents lists available at ScienceDirect

International Journal of Surgery Protocols

journal homepage: www.elsevier.com/locate/isjp

Protocols

Plexus anesthesia versus general anesthesia in patients for carotid endarterectomy with patch angioplasty: Protocol for a systematic review with meta-analyses and Trial Sequential Analysis of randomized clinical trials

M.S. Marsman [a,*], J. Wetterslev [b], F. Keus [c], D. van Aalst [d], F.G. van Rooij [e], J.M.M. Heyligers [f], F.L. Moll [g], A.Kh. Jahrome [h], P.W.H.E Vriens [f], G.G. Koning [i]

[a] Department of Vascular Surgery, Rijnstate Hospital, Arnhem, the Netherlands
[b] Copenhagen Trial Unit, Center for Clinical Intervention Research, Rigshospitalet Copenhagen University Hospital, Copenhagen, Denmark
[c] Department of Critical Care, University of Groningen, University Medical Center Groningen, the Netherlands
[d] Department of Anaesthesiology, Radboud University Medical Center Nijmegen, Nijmegen, the Netherlands
[e] Department of Neurology, Medical Center Leeuwarden, Leeuwarden, the Netherlands
[f] Department of Vascular Surgery, Elisabeth-TweeSteden Hospital, Tilburg, the Netherlands
[g] Department of Vascular Surgery, University Medical Center Utrecht, Utrecht, the Netherlands
[h] Department of Vascular Surgery, Medical Center Leeuwarden, Leeuwarden, the Netherlands
[i] Department of Vascular Surgery, Ikazia Hospital, Rotterdam, the Netherlands

ARTICLE INFO

Article history:
Received 3 November 2019
Received in revised form 21 December 2019
Accepted 23 December 2019
Available online 17 January 2020

ABSTRACT

Introduction: Traditional carotid endarterectomy is considered to be the standard technique for prevention of a new stroke in patients with a symptomatic carotid stenosis. Use of plexus anesthesia or general anesthesia in traditional carotid endarterectomy is, to date, not unequivocally proven to be superior to one other. A systematic review is needed for evaluation of benefits and harms to determine which technique, plexus anesthesia or general anesthesia is more effective for traditional carotid endarterectomy in patients with symptomatic carotid stenosis.

Methods and outcomes: The review will be conducted according to this protocol following the recommendations of the 'Cochrane Handbook for Systematic Reviews' and reported according to Preferred Reporting Items for Systematic Reviews and Meta-Analyses. Randomized Clinical Trials comparing plexus anesthesia versus general anesthesia in traditional carotid endarterectomy will be included. Primary outcomes will be postoperative death and/ or stroke (<30 days) and serious adverse events. Secondary outcomes will be non-serious adverse events.

We will primarily base our conclusions on meta-analyses of trials with overall low risk of bias. We will use Trial Sequential Analysis to assist the evaluation of imprecision in Grading of Recommendations Assessment, Development and Evaluation. However, if pooled point-estimates of all trials are similar to pooled point-estimates of trials with overall low risk of bias and there is lack of a statistical significant interaction between estimates from trials with overall high risk of bias and trials with overall low risk of bias we will consider the Trial Sequential Analysis adjusted confidence interval precision of the estimate achieved in all trials as the result of our meta-analyses.

Ethics and dissemination: The proposed systematic review will collect and analyze secondary data from already performed studies therefore ethical approval is not required. The results of the systematic review will be disseminated by publication in a peer-review journal and submitted for presentation at relevant conferences.

© 2020 The Author(s). Published by Elsevier Ltd on behalf of Surgical Associates Ltd. This is an open access article under the CC BY-NC-ND license (http://creativecommons.org/licenses/by-nc-nd/4.0/).

* Corresponding author at: M.D. Resident in Surgery, Rijnstate Hospital, Wagnerlaan 55, 6815 AD Arnhem, The Netherlands.

E-mail addresses: mmarsman@rijnstate.nl (M.S. Marsman), wetterslev@ctu.dk (J. Wetterslev), f.keus@umcg.nl (F. Keus), dennis.vanaalst@radboudumc.nl (D. van Aalst), frank.van.rooij@znb.nl (F.G. van Rooij), j.heyligers@etz.nl (J.M.M. Heyligers), f.l.moll@umcutrecht.nl (F.L. Moll), a.k.jahrome@znb.nl (A.Kh. Jahrome), pwhe.vriens@etz.nl (P.W.H.E Vriens), g.koning@ikazia.nl (G.G. Koning).

https://doi.org/10.1016/j.isjp.2019.12.002
2468-3574/© 2020 The Author(s). Published by Elsevier Ltd on behalf of Surgical Associates Ltd.
This is an open access article under the CC BY-NC-ND license (http://creativecommons.org/licenses/by-nc-nd/4.0/).

1. Introduction

There is still controversy which type of anesthesia, plexus anesthesia (PA) or general anesthesia (GA), is best for carotid endarterectomy (CEA). Guidelines of both the European Society of Vascular Surgery and the Dutch Society for Vascular Surgery recommend that choice of anesthesia for carotid endarterectomy (PA or GA) be left to the surgical team's preference [1–3]. Patients preferences or instruct ability could also play a role in the choice of technique.

The technique of CEA is previously described [4]. When a patient receives GA, the patient receives an opioid, muscle relaxant and an intravenous anesthetic such as propofol followed by intubation and mechanical ventilation [5]. When a patient receives PA a local anesthetic will be used e.g. ropivacaine. The patient is in a supine position facing away from the side of the surgery. The anesthetic will be put in place with guidance of anatomical landmarks or with help of an ultrasound by an anesthesiologist. Prior to the injection of the anesthetic lidocaine–prilocaine is applied to numb the skin. After skin disinfection the needle will be put in place at the level of the carotid bifurcation nearby the carotid sheath and a depot of the anesthetic will be placed superiorly and inferiorly along the posterior border of the sternocleidomastoid muscle. The carotid sheath can be numbed using ultra sound by the anesthesiologist of during surgery by the vascular surgeon.

Next to the plexus anesthesia, sedation can be considered to keep the patient comfortable. This sedation may consist of Dexmedetomidine, the first 10 minutes at 1 mcg/kg/h, after 10 min around 1/3 of the dosage guided by the heart frequency and or blood pressure. Simultaneously remifentanil is used at 0.1 mcg/kg/min, and after 10 min the dosage is continued at 0.05 lowered by 50%. Noradrenaline can be used to keep the blood pressure within its desired range to keep the brain adequately perfused.

Each type of anesthesia has its (dis)advantages. PA allows real-time monitoring compared to using Transcranial Doppler (TCD) and/or Electroencephalography (EEG) monitoring with GA. TCD and/or EEG monitoring are not perfect and assessment may be normal in 6% to 30% of those who develop neurological signs and abnormal in 3% to 11% of those who do not develop signs of ischemia [6]. Another advantage of PA is that the awake state of the patient does not impair the blood pressure regulation in contrast to GA, which may lower the risk of a periprocedural stroke [7]. PA is associated with a lower incidence of shunt placement during carotid endarterectomy [8]. The use of a shunt can prevent a perioperative ischemic event. However, it can cause damage to the arterial wall and/or cause an ischemic event [6]. Patients may have less post procedural pain compared with those after GA [9]. The main disadvantage of PA is conversion to GA, which can be necessary when the patient experiences too much pain. PA could also numb the phrenic nerve which can lead to intubation of patients with an already impaired pulmonary function. Other reasons for conversion to general anesthesia can be e.g. claustrophobia, airway obstruction due to cervical hematoma, loss of consciousness at carotid clamping, and shunt-related complications [10]. incidences of operative complications, such as local hemorrhage, cranial nerve damage, and pulmonary complications have been reported for both PA and GA and showed no differences [11].

Preventive management of (a)symptomatic carotid artery stenosis includes antiplatelet therapy, statins, antihypertensive therapy, diabetic control, as well as lifestyle modifications [11–14]. When a patient shows symptoms, different operation techniques are available and described in literature such as carotid endarterectomy with primary closure, eversion technique and traditional carotid endarterectomy with patch closure. Carotid endarterectomy with patch angioplasty is the preferred guideline treatment for patients with symptomatic stenosis of the carotid artery [14,16], primarily based on the European Carotid Surgery Trial (ECST) and the North American Symptomatic Carotid Endarterectomy Trial (NASCET) [1,4,17,18].

A multicenter, randomized clinical trial (RCT) included 3523 operations in 3523 symptomatic and asymptomatic patients and compared PA with GA and concluded that there was no significant difference between the two techniques for stroke (including retinal infarction), myocardial infarction, and death between randomization and 30 days after surgery [7]. However, the observed difference or lack of difference may or may not be affected by several confounding factors and/or differential use of co-interventions, such as the use of different surgical techniques, selected use of shunting, and variations in materials used for patching [18,20].

Previous conducted systematic reviews with meta-analysis of the randomized trials showed that there was no statistically significant difference between the PA and GA groups in the proportion of patients who had a stroke, died, or had a myocardial infarction within 30 days of carotid endarterectomy [10,21]. These reviews were conducted without Trial Sequential Analysis (TSA). To confirm or reject meta-analysis results we will add and TSA and include Grading of Recommendations Assessment, Development and Evaluation (GRADE) assessments of the evidence. We also try to reduce clinical heterogeneity by comparing only technique (PA) with one other technique (GA) in patients for carotid endarterectomy with patch angioplasty and also reduce the risk for random error.

To determine which technique, PA or GA is more effective for carotid endarterectomy with patch angioplasty in symptomatic carotid stenosis from the patients' perspective, it is important that all available evidence is evaluated according to the risks of errors in a systematic review in line with the Cochrane Handbook for Systematic Reviews of Interventions [21,23]. Therefore, an updated systematic review with meta-analyses is needed.

1.1. Objective

The objective is to conduct a systematic review with meta-analysis and Trial Sequential Analysis of randomized clinical trials, evaluating the benefits and harms of plexus anesthesia versus general anesthesia in carotid endarterectomy with patch angioplasty according to a pre-published protocol based on aspects of the *Cochrane Handbook for Systematic Reviews of Interventions* [22].

2. Methods

This review will be conducted according to this protocol, registered at PROSPERO CRD42019139913 [24] based on aspects of the recommendations of the 'Cochrane Handbook for Systematic Reviews of interventions' [22] and will be reported according to the Preferred Reporting Items for Systematic Reviews and Meta-Analyses (PRISMA) statement (www.prisma-statement.org) [25].

2.1. Studies

According to the current guideline [1,17,18] patients with a symptomatic stenosis ($\geq$50 – $\leq$99%) of the carotid artery will be considered. Only trials which evaluate plexus anesthesia versus general anesthesia in carotid endarterectomy with patch angioplasty in adult patients ($\geq$18 years) will be included [3]. Trials will be considered irrespective of language, blinding, outcomes, or publication status.

2.2. Experimental intervention

Plexus anesthesia (PA) in carotid endarterectomy with patch angioplasty.

2.3. Control intervention

General anesthesia (GA) in carotid endarterectomy with patch angioplasty.

2.4. Hypothesis

We want to test the null-hypothesis that there is no difference between the two treatments (H0: RRR = 0.00% or RR = 1.00) as well as both the alternative hypotheses (H1a and H1b) that there is a difference (H1a of a 10% RRR or H1b of a 15% RRR) between plexus anesthesia (PA) and general anesthesia (GA) in patients with a symptomatic carotid lesion. We think that patients operated with plexus anesthesia will do better because the neurological status of the patient can be monitored in real time compared with patients operated with general anesthesia in which the surgeon depends on a derived monitoring through TCD and EEG.

2.5. Outcomes

The outcome measures will be graded from the patients' perspective (GRADE working group 2008, Fig. 1) [26]. Examples of serious adverse events: stroke, bleeding, persisting neurological deficits, myocardial infarction, conversion PA to GA due to any cause, patients developing airway obstruction or phrenic nerve palsy and hypertension in need for (intravenous) medication.

2.6. Primary outcomes

- Proportion of participants who suffered death (<30 days).
- Proportion of participants with postoperative stroke (<30 days).
- Proportion of participants with one or more serious adverse events; which is defined as: any untoward medical occurrence that results in death, is life threatening, requires hospitalization or prolongation of existing hospitalization, results in persistent or significant disability or incapacity (or is a congenital anomaly or birth defect) [27].

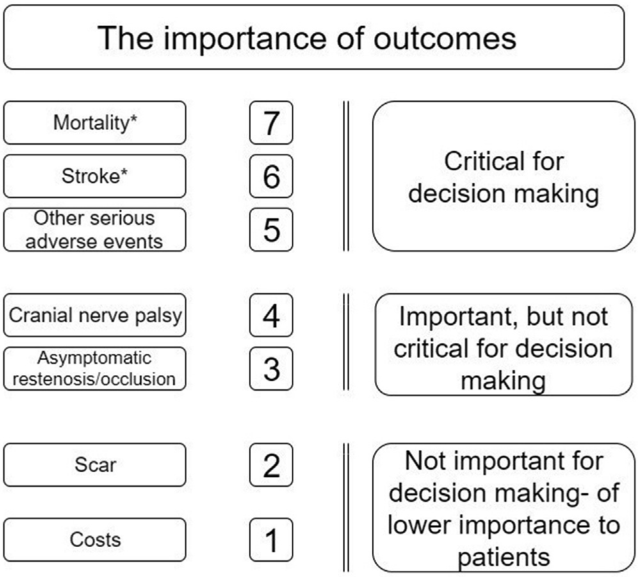

Fig. 1. Outcomes prioritized according to importance to patients undergoing carotid surgery for symptomatic carotid stenosis (GRADE 2008) [26] *<30 days.

2.7. Secondary outcomes

- Proportion of participants with one or more non-serious adverse events: any untoward medical occurrence in a participant that does not meet the above criteria for a serious adverse event is defined as a non-serious adverse event [27].
- Costs: hospitalization duration, duration of surgical procedure, ICU admission (e.g. blood pressure management).

2.8. Exploratory outcomes

- Separately reported serious adverse events.
- Separately reported non-serious adverse events.

The number of patients with one or more complications will be evaluated rather than the numbers of events, depending on the availability of data.

2.9. Search strategy

The Cochrane Central Register of Controlled Trials (CENTRAL) in the Cochrane Library, PubMed/MEDLINE and EMBASE will be searched. References of the identified trials will be searched to identify any further relevant randomized clinical trials. The search strategies are provided in the appendix. Searches will include MeSH descriptors such as "Clinical Trials", "carotid endarterectomy", "plexus", "carotid artery disease", "anesthesia", "patch". We will also search online trial registries such as ClinicalTrials.gov (https://clinicaltrials.gov/), European Medicines Agency (EMA) (www.ema.europa.eu/ema/), WHO International Clinical Trial Registry Platform (www.who.int/ictrp), and the Food and Drug Administration (FDA) (www.fda.gov) for ongoing or unpublished trials. In addition, we plan to search Google Scholar (https://scholar.google.nl/) using the terms: anesthesia and/or plexus and/or local anesthesia and/or carotid and/or endarterectomy in the title of the abstract/paper.

2.10. Data collection

Two authors will perform screening and select the trials for inclusion, independently. Excluded trials and studies will be listed with their reasons for exclusion. When disagreements should occur, a third author will be approached to reconcile. The authors will extract the following data when available: type of anesthesia, trial characteristics (year and language of publication, country in which the trial was conducted, year of conduction of the trial, single or multicenter trial, number of patients), patient characteristics (inclusion and exclusion criteria, mean age, mean body mass index and gender, smoking, diabetes mellitus, use of statin and platelet inhibitors), intervention characteristics (general anesthesia, plexus anesthesia, closure by type of patch, use of shunting), co-interventions (conversion to general anesthesia, perioperative transcranial Doppler monitoring, perioperative carotid pressure measurement, electroencephalographic monitoring) and the outcome measures evaluated. If there are any unclear or missing data, the corresponding authors of the individual trials will be contacted, at least twice, for clarification.

2.11. Risk of bias assessment

Two authors will assess the risks of bias, without masking for trial names, according to the Cochrane Handbook for Systematic Reviews of Interventions [22], including the domains of generation

of the allocation sequence, allocation concealment, blinding of participants, personnel, and outcome assessors, incomplete outcome data, selective outcome reporting, and bias risks such as vested interests (financial interest, academical interest or other parties such as the medical industry). Risk of bias components will be scored as low, unclear, or high risk of bias.

2.12. Sequence generation

- Low risk of bias: The method used (e.g. central allocation) is unlikely to induce bias on the final observed effect, such as:
  - referring to a random number table;
  - using a computer random number generator;
  - coin tossing;
  - shuffling cards or envelopes;
  - throwing dice;
  - drawing of lots.
- Unclear risk of bias: Insufficient information to assess whether the method used is likely to introduce confounders.
- High risk of bias: The method is improper and likely of introduce confounding, e.g. based on date of admission, or record number, or by odd or even date of birth.

2.13. Allocation concealment

Some aspects of the conduct of randomized trials, particularly blinding, are associated with a modest exaggeration of treatment effects on average, but there is little evidence that the average bias differs according to whether the outcome was subjectively or objectively assessed. However, lack of blinding in trials with subjective outcomes leads to increased heterogeneity and hence unpredictable bias in effect estimates. As far as possible, clinical and policy decisions should be cautious when they are based on trials in which blinding was not reported or not feasible and outcome measures were subjectively assessed [28].

- Low risk of bias: Participants and investigators enrolling participants could not foresee assignment because one of the following, or an equivalent method, was used to conceal allocation:
- central allocation (including telephone);
- web-based and pharmacy-controlled randomization;
- sequentially numbered drug containers of identical appearance;
- sequentially numbered, opaque, sealed envelopes.
- Unclear risk of bias: Insufficient information to permit judgement of 'Low risk' or 'High risk'. This is usually the case if the method of concealment is not described or not described in sufficient detail to allow a definite judgement.
- High risk of bias: Participants or investigators enrolling participants could possibly foresee assignments and thus introduce selection bias, such as allocation based on:
  - an open random allocation schedule;
  - assignment envelopes were used without appropriate safeguards;
  - alternation or rotation;
  - date of birth;
  - case record number;
  - any other explicitly unconcealed procedure.

2.14. Blinding of participants and personnel

In surgical procedures it is impossible to blind the surgeon who performs the procedure of CEA, while it is possible to blind the caregivers responsible for postoperative care as well as the patients [29]. In this type of comparison with PA and GA no blinding is possible. For this domain we cannot consider the surgeon, caregivers and patients, so a certain risk of bias will inevitably be present when evaluating surgical procedures.

- Low risk of bias: No blinding or incomplete blinding, but the review authors judge that the outcome is not likely to be influenced by lack of blinding or blinding of participants and key study personnel ensured, and it is unlikely that the blinding could have been broken.
- Unclear risk of bias: Insufficient information to permit judgement of 'Low risk' or 'High risk', or the study did not address this outcome.
- High risk of bias: No blinding or incomplete blinding, and the outcome is likely to be influenced by lack of blinding or blinding of key study participants and personnel attempted, but likely that the blinding could have been broken, and the outcome is likely to be influenced by lack of blinding.

2.15. Blinding of outcome assessment

- Low risk of bias: No blinding of outcome assessment, but the review authors judge that the outcome measurement is not likely to be influenced by lack of blinding or blinding of outcome assessment is ensured, and it is unlikely that the blinding could have been broken.
- Unclear risk of bias: Insufficient information to permit judgement of 'Low risk', or 'High risk' or the study did not address this outcome.
- High risk of bias: No blinding of outcome assessment, and the outcome measurement is likely to be influenced by lack of blinding, or blinding of outcome assessment, but likely that the blinding could have been broken, and the outcome measurement is likely to be influenced by lack of blinding.

2.16. Incomplete outcome data

- Low risk of bias:
- no missing outcome data;
- reasons for missing outcome data unlikely to be related to true outcome (for survival data, censoring unlikely to be introducing bias);
- missing outcome data balanced in numbers across intervention groups, with similar reasons for missing data across groups;
- for dichotomous outcome data, the proportion of missing outcomes compared with observed event risk is not enough to have a clinically relevant impact on the intervention effect estimate;
- for continuous outcome data, plausible effect size (difference in means or standardized difference in means) among missing outcomes is not enough to have a clinically relevant impact on observed effect size;
- missing data have been imputed using appropriate methods.
- Unclear risk of bias: Insufficient reporting of attrition/exclusions to permit judgement of 'Low risk' or 'High risk' (e.g. number randomized not stated, no reasons for missing data provided) or the study did not address this outcome.
- High risk of bias:
- reason for missing outcome data likely to be related to true outcome, with either imbalance in numbers or reasons for missing data across intervention groups;
- for dichotomous outcome data, the proportion of missing outcomes compared with observed event risk enough to induce clinically relevant bias in intervention effect estimate;

- for continuous outcome data, plausible effect size (difference in means or standardized difference in means) among missing outcomes enough to induce clinically relevant bias in observed effect size;
- 'as-treated' analysis done with substantial departure of the intervention received from that assigned at randomization;
- potentially inappropriate application of simple imputation.

2.17. Selective outcome reporting

- Low risk of bias: The study protocol is available and all the studies pre-specified (primary and secondary) outcomes that are of interest in the review have been reported in the pre-specified way, or the study protocol is not available but it is clear that the published reports include all expected outcomes, including those that were pre-specified.
- Unclear risk of bias: Insufficient information to permit judgement of 'Low risk' or 'High risk'. It is likely that the majority of studies will fall into this category.
- High risk of bias:
- not all of the studies pre-specified primary outcomes have been reported;
- one or more primary outcomes is reported using measurements, analysis methods or subsets of the data (e.g. subscales) that were not pre-specified;
- one or more reported primary outcomes were not pre-specified (unless clear justification for their reporting is provided, such as an unexpected adverse effect);
- one or more outcomes of interest in the review are reported incompletely so that they cannot be entered in a meta-analysis;
- the study report fails to include results for a key outcome that would be expected to have been reported for such a study.

2.18. Other bias

- Low risk of bias: The study appears to be free of other sources of bias.
- Unclear risk of bias: There may be a risk of bias, but there is either insufficient information to assess whether an important risk of bias exists or insufficient rationale or evidence that an identified problem will introduce bias.
- High risk of bias: There is at least one important risk of bias.

2.19. Overall risk of bias

Trials were classified as trials with low overall risk of bias if all risk of bias domains were scored as having low risk of bias. If one or more of the bias domains were scored as unclear or high risk of bias, the trial was considered to have high overall risk of bias. Trials classified as low risk of bias in all domains of sequence generation, allocation concealment, blinding, incomplete data, selective outcome reporting, source of funding and other potential risks of bias will be considered trials at overall low risk of bias. Trials with one or more of these domains scored as unclear or high risk of bias will be considered trials at overall high risk of bias [22,28,30].

2.20. Statistical methods

Meta-analyses will be performed according to the Cochrane Handbook for Systematic Reviews of Interventions [22]. The software package Review Manager (RevMan) Version 5.3 will be used [31]. Significance levels will be adjusted due to multiplicity of several outcomes. The results of each outcome will require an adjusted statistical significance level (threshold). An alfa of respectively $(0.05/((1 + 3)/2) =)$ 0.025 will be used for the primary and 0.033 for the secondary outcomes to keep the family wise error rate (FWER) below 0.05 [31,33]. For exploratory outcomes, we will consider a *p*-value less than 0.05 as significant, because we view these outcomes as only hypothesis-generating outcomes. For dichotomous variables, the risk ratio (RR) with TSA-adjusted confidence intervals (CI) will be calculated. For continuous variables, the mean difference (MD) with TSA-adjusted CI will be calculated or the standardized mean difference (SMD) with 95% CI will be calculated.

For the outcome of SAE we plan to estimate the proportion of patients with one or more SAE in each group and to analyze this outcome in a binary meta-analysis. However, as we anticipate the reporting of SAEs in trials to vary considerably we plan to do two analyses and to avoid multiple counts of SAE in the same patients (SAE counting is not a statistical independent outcome):

1) The cumulated SAE analysis: Assuming that only one SAE is reported per patient. We will summarize all reported SAE in each trial and calculate the proportion of summed SAE divided with number of randomized patients in the experimental and control intervention group, the number of patients in each group will constitute a maximum of SAEs (maximum proportion = 1.00).

2) The highest proportion of SAEs registered: we will analyze the most frequent SAE in each included trial as if it represents the total number of patients with SAEs in the experimental and control intervention group assuming that if a patient don't get the SAE with the highest proportion in the trial they don't get another SAE either. Being aware that none of these intervention effect estimates (1 or 2) are exactly correct we will discuss differences between the effect of the experimental vs the control intervention on the proportion of patients with one or more SAEs.

The impact of attrition bias will be explored using best/ worst and worst/ best case scenarios: a best/ worst case scenario is one where all patients lost to follow-up in the intervention group are supposed to have survived while all patients lost to follow-up in the control intervention group have died. A worst/best case scenario is the reverse.

Heterogeneity will be explored by chi-squared test with significance set at *p*-value of 0.10, and the quantity of heterogeneity will be measured by I^2. We will conduct both random-effects model and fixed-effect model meta-analyses. In case of discrepancies the results of both models will be presented and we will primarily stress the result of the model with the result closest to null effect due to principle of cautiousness [33]. The analyses will be performed on an intention-to-treat basis whenever possible.

A funnel plot will be used to explore small trial bias and to use asymmetry in funnel plot of trial size against treatment effect to assess this bias. Begg's and Egger's tests will be used to test for asymmetry in funnel plots [34].

2.21. Trial Sequential analyses (TSA)

Meta-analyses may result in type-I errors and type-II errors due to an increased risk of random error when sparse data are analyzed and due to repeated significance testing when a cumulative meta-analysis is updated with new trials [34,36]. To assess the risk of type-I and type-II errors, TSA will be used. The vast majority of meta analyses (nearly 80%) in Cochrane systematic reviews have less than the required information size to conclude on a 30% relative risk reduction (RRR) and less than 2% have sufficient power to conclude on a 10% RRR [36–39].

TSA combines information size estimation for meta-analysis (cumulated sample size of included trials) with an adjusted threshold for statistical significance of meta-analysis [34,36,40]. The latter, called trial sequential monitoring boundaries (TSMB), reduce type-I errors. In TSA the addition of each trial in a cumulative meta-analysis is regarded as an interim analysis and helps to

clarify whether additional trials are needed or not. The idea in TSA is that when the cumulative z-curve crosses the TSMB, a sufficient level of evidence has been reached and no further trials may be needed. If the z-curve does not cross the boundary of benefit and the required information size has not been reached, there may be insufficient evidence to reach a conclusion [34,36,41,42]. TSA can also be used for the evaluation of type II errors, that is, to evaluate whether further randomized trials are futile to show or discard the anticipated intervention effect (RRR or MD). This happens when the cumulative z-curve does cross the TSMBs for futility. TSA will be applied since it controls the risks of type-I and type-II errors in a cumulative meta-analysis and may provide important information on how many more patients need to be included in further trials. The information size will be calculated as diversity-adjusted required information size (DARIS) [43]. We will do the primary analysis calculating the DARIS based on an a priori anticipated intervention effect of a 10% RRR which is close to a minimal important difference. We will conduct sensitivity analyses for a 15% RRR as well as the RRR suggested by the meta-analysis of the included trials [44]. If the estimated Diversity of the meta-analysis is 0%, a sensitivity analysis with TSA using a Diversity of 25% will be conducted. TSA will be performed on all outcomes. The required information size for primary outcomes will be calculated based on an a priori RRR of 10% and appropriately adjusted for diversity according to an overall type-I error of 2.5% for the co-primary outcomes and 3.3% for the secondary outcomes to account for a family wise error (FWER) of 5% all in all, we will use a power of 90% considering sparse data and repetitive testing [43]. For secondary outcomes the DARIS will be calculated using a power of 90% [43]. As a sensitivity analysis, the DARIS will be calculated using the estimated intervention effect from the trials at low risk of bias in a conventional meta-analysis. If the required information size is surpassed for the TSA using the estimated intervention effect in the conventional meta-analysis or a TSMB is crossed a TSA with an anticipated intervention effect equal to the confidence limit closest to the null effect in the effect estimate from the conventional meta-analysis will be performed.

The TSAs will be conducted using the control event proportion calculated from the unweighted control event proportion from the control groups of the actual meta-analyses.

2.22. Subgroup analyses

The following subgroup analysis will be performed:

Trials at overall low risk of bias (all except blinding of surgeons scored as low risk of bias) compared to trials at high overall risk of bias (two or more of the bias domains (including blinding of surgeons) scored as unclear or high risk).

3. Grade

We will use summary of findings tables to summarize the results of the trials with overall low risk of bias and for all trials, separately. Reasons for downgrading the quality of the available evidence are: risk of bias evaluation of the included bias domains, publication bias, heterogeneity, imprecision, and indirectness (e.g. length of stay is a surrogate outcome measure) [44–47]. We will compare the imprecision assessed according to GRADE with that of TSA [48].

3.1. Patient and public involvement

Patients and/ or public were not involved in this study.

3.2. Ethics and dissemination

The proposed systematic review will collect and analyze secondary data from already performed studies therefore ethical approval is not required. The results of the systematic review will be disseminated by publication in a peer-review journal and submitted for presentation at relevant conferences.

3.3. Protocol timeline

First registration of the protocol at Prospero CRD42019139913 in August 2019.

Proposed date of starting the search: 1st of January 2020. Proposed date of finishing the review is the 31st of March 2020.

Ethical approval

None.

Funding

None.

Author contribution

MSM is the first author of the protocol. MSM and GGK managed the first draft of this manuscript and coordinated the contributions of coauthors. Contributors JW, PWHEV, DA, FGR, JMMH, FLM, AkhJ, FK, and GGK contributed to the design of the study and revised the paper critically. JW, FK, and GGK provided professional and statistical support. All authors read and approved the final version of the manuscript. GGK was initiator and supervisor.

Conflict of interest statement

JW is a member of the taskforce at Copenhagen Trial Unit to develop theory and software doing TSA, presently available as freeware at www.ctu.dk/tsa.

Guarantor

MSM and GGK.

Research registration number

None.

Acknowledgement

The authors would like to thank Mrs. L.W.M. Boerboom, MSc, medical information specialist (Medical Library, Elisabeth-TweeSteden Hospital, Tilburg, the Netherlands) for her assistance.

Appendix A. Supplementary data

Supplementary data to this article can be found online at https://doi.org/10.1016/j.isjp.2019.12.002.

References

[1] A. Naylor, Management of Atherosclerotic Carotid and Vertebral Artery Disease: 2017 Clinical Practice Guidelines of the European Society for Vascular Surgery (ESVS), Eur. J. Vasc. Endovasc. Surg. [Internet] 55 (1) (2017) 1–79, Available from: http://dx.doi.org/10.1016/j.ejvs.2017.06.021.

[2] C.D. Liapis, S.P.R.F. Bell, D. Mikhailidis, J. Sivenius, A. Nicolaides, Fernandes e Fernandes J, ESVS, et al., Guidelines. Invasive treatment for carotid stenosis: indications, techniques, Eur. J. Vasc. Endovasc. Surg. 37 (4 Suppl.) (2009) 1–19.
[3] Nederlandse Vereniging voor Neurologie en het Kwaliteitsinstituut voor de Gezondheidszorg CBO. Diagnostiek, behandeling en zorg voor patiënten met een beroerte [Internet]. Richtlijn Diagnostiek, behandeling en zorg voor patiënten met een beroerte. 2008 [cited 2017 Dec 2]. Available from: http://med-info.nl/Richtlijnen/Geriatrie/Beroerte.pdf.
[4] M.S. Marsman, J. Wetterslev, A.K. Jahrome, C. Gluud, F.L. Moll, A. Karimi, et al., Carotid endarterectomy with primary closure versus patch angioplasty in patients with symptomatic and significant stenosis: protocol for a systematic review with meta-analyses and trial sequential analysis of randomised clinical trials, BMJ Open (2019) 1–7, https://doi.org/10.1136/bmjopen-2018-026419.
[5] D. Wong, A. Dallaire, K. Singh, P. Madhusan, T. Jackson, M. Singh, et al., High-flow nasal oxygen improves safe apnea time in morbidly obese patients undergoing general anesthesia: a randomized controlled trial, Anesth. Analg. 129 (4) (2019) 1130–1136.
[6] K. Rerkasem, P.M. Rothwell, Routine or selective carotid artery shunting for carotid endarterectomy (and different methods of monitoring in selective shunting), Cochrane Database Syst. Rev. [Internet] (4) (2009), CD000190 Available from: https://doi.org/10.1002/14651858.CD000190.pub2.
[7] Trial G, Group C, General anaesthesia versus local anaesthesia for carotid surgery (GALA): a multicentre, randomised controlled trial, Lancet [Internet] 372 (9656) (2008) 2132–2142, Available from: http://dx.doi.org/10.1016/S0140-6736(08)61699-2.
[8] K. Watts, P.H. Lin, R.L. Bush, S. Awad, et al., The impact of anesthetic modality on the outcome of carotid endarterectomy, Am. J. Surg. 188 (2004) 741–747, https://doi.org/10.1016/j.amjsurg.2004.08.048.
[9] W. Do, A.-R. Cho, E.-J. Kim, et al., Ultrasound-guided superficial cervical plexus block under dexmedetomidine sedation versus general anesthesia for carotid endarterectomy: a retrospective pilot study, Yeungnam Univ. J. Med. 35 (1) (2018) 45–53, https://doi.org/10.12701/yujm.2018.35.1.45.
[10] L. Pasin, P. Nardelli, G. Landoni, G. Cornero, S. Magrin, Y. Tshomba, R. Chiesa, A. Zangrillo, Examination of regional anesthesia for carotid endarterectomy, J. Vasc. Surg. 62 (3) (2015) 631–634.e1, https://doi.org/10.1016/j.jvs.2015.03.074.
[11] K. Rerkasem, R. Bond, R. Pm, Local versus general anaesthesia for carotid endarterectomy (Review), Cochrane Collab. [Internet] (2) (2005), Available from: https://doi.org/10.1002/14651858.CD000126.pub2.
[12] G. Raman, D. Moorthy, N. Hadar, I.J. Dahabreh, T.F. O'Donnell, Management Strategies for asymptomatic Carotid Stenosis: a systematic review and meta-analysis, Ann. Intern. Med. 158 (9) (2013) 676–685.
[13] A.L. Abbott, Medical (nonsurgical) intervention alone is now best for prevention of stroke associated with asymptomatic severe carotid stenosis: Results of a systematic review and analysis, Stroke 40 (10) (2009) 573–584.
[14] J. Constantinou, P. Jayia, G. Hamilton, Best evidence for medical therapy for carotid artery stenosis, J. Vasc. Surg. 58 (4) (2013) 1129–1139.
[15] C.S. Cina, C.M. Clase, R.B. Haynes, S. Orrapin, K. Rerkasem, Carotid endarterectomy for symptomatic carotid stenosis, Cochrane Database Syst. Rev. [Internet] (2) (2017), CD001081. Available from: 10.1002/14651858.CD001081.
[16] S. Bangalore, S. Kumar, J. Wetterslev, A.A. Bavry, C. Gluud, D.E. Cutlip, et al., Carotid artery stenting vs carotid endarterectomy: meta-analysis and diversity-adjusted trial sequential analysis of randomized trials, Arch Neurol. 68 (2) (2011) 172–184.
[17] C. Warlow, MRC European Carotid Surgery Trial: interim results for symptomatic patients with severe (70–99%) or with mild (0–29%) carotid stenosis, Lancet 337 (8752) (1991) 1235–1243.
[18] Collaborators NASCET, Beneficial effect of carotid endarterectomy in symptomatic patients with high-grade carotid stenosis, N. Engl. J. Med. 325 (7) (1991) 445–453.
[19] M. Orlicky, T. Hrbac, M. Sames, P. Vachata, A. Hejcl, D. Otahal, et al., Anesthesia type determines risk of cerebral infarction after carotid endarterectomy, J. Vasc. Surg. [Internet] 70 (1) (2018) 138–147, https://doi.org/10.1016/j.jvs.2018.10.066.
[20] V.V. Lomivorotov, V.A. Shmyrev, V.A. Nepomniashchikh, Regional versus general anesthesia for carotid endarterectomy: do we need another randomized trial?, J Cardiothorac. Vasc. Anesth. [Internet] (2018) 7–8, Available from: https://doi.org/10.1053/j.jvca.2018.09.007.
[21] A. Harky, J. Shi Kai, Chan, T. Ka Ming Kot, D. Sanli, R. Rahimli, Z. Belamaric, et al., General anaesthesia versus local anaesthesia in carotid endarterectomy: a systematic review and meta-analysis, J. Cardiothorac. Vasc. Anesth. (2019).
[22] J. Higgins, S. Green, Cochrane Handbook for Systematic Review of Intervention Version 5.1.0 [Internet], The Cochrane Collaboration (2011), Available from: www.Cochrane-handbook.org.
[23] F. Keus, J. Wetterslev, C. Gluud, et al., Evidence at a glance: error matrix approach for overviewing available evidence, BMC Med. Res. Methodol. 10 (2010) 90.
[24] M.S. Marsman, J. Wetterslev, F. Keus, D. Aalst van, F.G. Rooij van, J.M.M. Heyligers, et al., Plexus anesthesia versus general anesthesia in patients for carotid endarterectomy with patch angioplasty: protocol for a systematic review with meta-analyses and Trial Sequential Analysis of randomized clinical trials, Prospero [Internet] (2019), Available from: https://www.crd.york.ac.uk/prospero/display_record.php?RecordID=139913.
[25] D. Moher, A. Liberati, J. Tetzlaff, D.G.G.P. Altman, Preferred reporting items for systematic reviews and meta-analyses: the PRISMA statement, PLoS Med. 6 (7) (2009).
[26] G.H. Guyatt, A.D. Oxman, R. Kunz, G.E. Vist, Y. Falck-Ytter, H.J. Schünemann, et al., What is "'quality of evidence'" and why is it important to clinicians?, BMJ 336 (7651) (2008) 995–998
[27] International Conference on Harmonisation Expert Working Group, International conference on harmonization of technical requirements for registration of pharmaceuticals for human use. ICH harmonised tripartite guideline. Guideline for good clinical practic, Guidel. Good Clin. Pract. CFR ICH Guidel. 1 (1997).
[28] J. Savović, R.M. Turner, D. Mawdsley, H.E. Jones, R. Beynon, P.T. Julian, Association between risk-of-bias assesments and results of randomized trials in coachrane reviews: the ROBES Meta-Epidemiologic Study, Am. J. Epidemiol. 187 (5) (2018) 113–1122.
[29] K.S. Gurusamy, C. Gluud, D. Nikolova, B.R. Davidson, Assessment of risk of bias in randomized clinical trials in surgery, Br. J. Surg. 96 (4) (2009) 342–349.
[30] J. Higgins, R. Churchill, T. Lasserson, J. Chandler, D. Tovey, Update from the Methodological Expectations of Cochrane Intervention Reviews (MECIR) project, Cochrane Meth. (2012) 2–3.
[31] Review Manager (RevMan) [Internet], Copenhagen: The Nordic Cochrane Centre, The Cochrane Collaboration (2014), Available from: https://community.cochrane.org/help/tools-and-software/revman-5.
[32] J.C. Jakobsen, J. Wetterslev, T. Lange, C. Gluud, Editorial - Viewpoint: taking into account risks of random errors when analysing multiple outcomes in systematic reviews | Cochrane, Library. (2016) 2–7.
[33] J.C. Jakobsen, J. Wetterslev, P. Winkel, T. Lange, C. Gluud, Thresholds for statistical and clinical significance in systematic reviews with meta-analytic methods, BMC Med. Res. Methodol. 14 (1) (2014) 1–13.
[34] G.G. Koning, J. Wetterslev, C.J.H.M. van Laarhoven, F. Keus, The totally extraperitoneal method versus Lichtenstein's technique for inguinal hernia repair: a systematic review with meta-analyses and trial sequential analyses of randomized clinical trials, PLoS One 8 (1) (2013).
[35] J. Wetterslev, K. Thorlund, J. Brok, C. Gluud, Trial sequential analysis may establish when firm evidence is reached in cumulative meta-analysis, J. Clin. Epidemiol. 61 (1) (2008) 64–75.
[36] J. Brok, K. Thorlund, J. Wetterslev, C. Gluud, Apparently conclusive meta-analyses may be inconclusive - trial sequential analysis adjustment of random error risk due to repetitive testing of accumulating data in apparently conclusive neonatal meta-analyses, Int. J. Epidemiol. 38 (1) (2009) 287–298.
[37] R.M. Turner, S.M. Bird, J.P.T. Higgins, The impact of study size on meta-analyses: examination of underpowered studies in cochrane reviews, PLoS One 8 (3) (2013) 1–8.
[38] E.J. Mascha, Alpha, beta, meta: Guidelines for assessing power and Type I error in meta-analyses, Anesth Analg. 121 (6) (2015) 1430–1433.
[39] G. Imberger, K. Thorlund, C. Gluud, J. Wetterslev, False-positive findings in Cochrane meta-analyses with and without application of trial sequential analysis: an empirical review, BMJ Open 6 (8) (2016) e011890.
[40] K. Thorlund, P.J. Devereaux, J. Wetterslev, G. Guyatt, J.P.A. Ioannidis, L. Thabane, et al., Can trial sequential monitoring boundaries reduce spurious inferences from meta-analyses?, Int. J. Epidemiol. 38 (1) (2009) 276–286.
[41] J. Pogue, S. Yusuf, Overcoming the limitations of current meta-analysis of randomised controlled trials, Lancet 351 (9095) (1998) 47–52.
[42] J.M. Pogue, S. Yusuf, Cumulating evidence from randomized trials: Utilizing sequential monitoring boundaries for cumulative meta-analysis, Control Clin. Trials 18 (6) (1997) 580–593.
[43] J. Wetterslev, K. Thorlund, J. Brok, C. Gluud, Estimating required information size by quantifying diversity in random-effects model meta-analyses, BMC Med. Res. Methodol. 9 (2009) 1–12.
[44] J. Wetterslev, J.C. Jakobsen, C. Gluud, Trial sequential analysis in systematic reviews with meta-analysis, BMC Med. Res. Methodol. 17 (1) (2017) 1–18.
[45] G.H. Guyatt, A.D. Oxman, R. Kunz, J. Brozek, P. Alonso-Coello, D. Rind, et al., GRADE guidelines 6. Rating the quality of evidence – Imprecision, J. Clin. Epidemiol. 64 (12) (2011) 1283–1293.
[46] J. Savović, H.E. Jones, D.G. Altman, R.J. Harris, P. Juni, J. Pildal, et al., Influence of reported study design characteristics on intervention effect estimates from randomised controlled trials: combined analysis of meta-epidemiological studies, Health Technol. Assess (Rockv). 16 (35) (2012) 1–81.
[47] J. Savovic, H.E. Jones, D.G. Altman, R.J. Harris, J. Pildal, B. Als-nielsen, et al., Research and reporting methods influence of reported study design characteristics on intervention, Ann. Intern. Med. 157 (6) (2012) 429–438.
[48] G. Castellini, M. Bruschettini, S. Gianola, C. Gluud, L. Moja, Assessing imprecision in Cochrane systematic reviews: a comparison of GRADE and Trial Sequential Analysis, Syst. Rev. 7 (1) (2018) 1–10.
